# Comparing low-cost handheld autorefractors: A practical approach to measuring refraction in low-resource settings

**DOI:** 10.1371/journal.pone.0219501

**Published:** 2019-10-15

**Authors:** Arunika Agarwal, David E. Bloom, Vincent P. deLuise, Alyssa Lubet, Kaushik Murali, Srinivas M. Sastry

**Affiliations:** 1 Department of Global Health and Population, Harvard T.H. Chan School of Public Health, Boston, United States of America; 2 Department of Ophthalmology, Yale University School of Medicine, New Haven, Connecticut, United States of America; 3 Center for Education Policy Research, Harvard University Graduate School of Education, Cambridge, MA, United States of America; 4 Sankara Academy of Vision, Sankara Eye Hospital, Bangalore, Karnataka, India; 5 Bethesda Retina, Bethesda, Maryland, United States of America; Western University, CANADA

## Abstract

**Purpose:**

To compare and validate the accuracy and ease of use of handheld autorefractors against retinoscopic refraction by an ophthalmologist for assessing the visual acuity of older adults in India.

**Methods:**

190 patients were enrolled at the Sankara Eye Hospital in Bangalore, India, to undergo refraction using three different handheld devices—Retinomax (Nikon Inc., Japan), Netra (Eyenetra, Inc., USA), and QuickSee (PlenOptika, Inc., USA)—and the results were compared with cycloplegic retinoscopy and refraction done by an ophthalmologist. We analyzed the mean, standard deviation (S.D.), and Bland-Altman comparison of dioptric (D) power accuracy.

**Results:**

The difference between the handheld devices and subjective refraction for each device was: Retinomax (N = 186), mean -0.41 D, S.D. 2.14; Netra (N = 179), mean 0.61 D, S.D. 2.20; and QuickSee (N = 182), mean -0.05 D, S.D. 1.04.

**Conclusion:**

The QuickSee and the Retinomax may be used successfully as refraction screening tools in epidemiologic studies of adults in India and as diagnostic tools in low-resource settings.

## Introduction

Despite multinational and multisystem efforts to combat global visual impairment and blindness, projections of visual loss are staggering, and cases of visual impairment appear to be accelerating. The Vision Loss Expert Group reports that, taking into account current levels of global population growth and population aging, more than 38 million individuals could be blind by 2020 and nearly 115 million by 2050.[1] The social and economic consequences of visual impairment are far-reaching, including poor health outcomes and poor quality of life, affecting individuals, households, and whole communities. [2][3] Relative to the non-visually impaired counterparts, visually impaired individuals are three times as likely to be unemployed, three times as likely to be involved in a motor vehicle collision, three times as likely to suffer from depression and anxiety, and twice as likely to fall while walking.[4] Women and socioeconomically disadvantaged groups have higher rates of blindness than men and higher-income individuals.[1,5] From a global economic perspective, the annual health system cost of recognizing and preventing vision loss and treatable blindness has been estimated at US$2.3 trillion.[6] Brown et al. show that interventions to prevent vision loss yield great returns on investment, especially in lower-income countries.[7]

Globally, 80% of all vision impairment can be prevented or corrected.[4] With more than 53% of visual impairment caused by uncorrected refractive errors (visual acuity less than 20/20 feet, 6/6 meter, 1.00 decimal, or 0.0 LogMAR—LogMAR is an acronym for Logarithm of the Minimum Angle of Resolution [MAR]-–if an individual can resolve details as small as 1 minute of visual angle, it corresponds to a LogMAR of 0, since the base-10 logarithm of 1 is 0),[4] efforts to screen and correct those affected would have high rates of return in reducing the global burden of vision impairment. Unfortunately, traditional delivery of refraction diagnostic services relies principally on deploying highly trained eye care providers in the field (ophthalmic technicians, optometrists, or ophthalmologists). Higher-income nations have the resources to buy and use costly equipment, such as conventional autorefractors, and to provide refraction diagnostic services that are streamlined to handle large volumes of patients. These costly devices can be used by non-eye-specialist healthcare practitioners and layperson screeners.[8] However, healthcare delivery systems in developing nations rarely have either the financial resources to obtain this technology or the trained personnel to screen large volumes of patients for refractive errors and other vision conditions.

A potential solution to this problem lies in the recent development of several relatively inexpensive, easy-to-operate handheld technologies designed to measure refraction. These technologies are lightweight and self-contained, with attachments similar to those on smartphones that provide real-time data and printouts. Such devices may be particularly useful in large-scale population surveys, as trained volunteers can operate them and their portability allows them to be easily transported from one field location to another to conduct in-home health examinations. Due to such advantages, these technologies have the potential to be valuable tools that can be integrated into research and extend healthcare coverage to remote areas.

In an effort to validate and compare three of these portable, lower-cost devices with the “gold standard” of subjective refraction, we performed a prospective study of older adult patients presenting to an eye hospital in India.

## Methods

The study was performed in accordance with the tenets of the Declaration of Helsinki and was approved by the Institutional Review Board (IRB) and Office of Human Research Administration of Harvard University, Boston, USA, and the Sankara Eye Hospital Ethics Board, Bangalore, India.

The study enrolled 205 patients presenting for eye exams to the Sankara Eye Hospital in Bangalore, India, in August 2016. An informed written consent was obtained and translators were provided as needed. Since the objective of the study was to identify a handheld auto-refractor that could be used to measure visual acuity among older adults in a field setting, the study excluded individuals under the age of 40 years, those with active signs of ocular infection/conjunctivitis or acute eye trauma, and those physically or cognitively unable to be tested using the handheld devices. However, all the patients, whether included in or excluded from the study, received appropriate care from Sankara Eye Hospital.

All subjects underwent refraction via four methods, including three handheld autorefraction devices—(1) Retinomax (Nikon, Japan), (2) Netra, 2016 model (Eyenetra, USA), and (3) QuickSee (PlenOptika, USA)—and (4) a retinoscopy/refraction noncycloplegic and cycloplegic exam by an ophthalmologist (collectively referred to as “subjective refraction” in this paper). A supervising ophthalmologist and trained optometry personnel performed all exams. To avoid test bias, all subjects and personnel were masked from the results and interchanged at exam stations. All exams were done on the same consult visit day for each study subject. Sphere, cylinder, and axis of cylinder were recorded for all devices. For this study, we have used spherical equivalent to define refractive error, which was calculated mathematically by adding sphere power and half of cylinder power. Subjects in poor compliance with the exam procedures were excluded. All statistical analyses, including Bland-Altman comparisons, were performed with STATA 14 (StataCorp LLC, College Station, TX).

## Results

Of the 190 patients who completed the study, nine were excluded from the Retinomax, 16 were excluded from the Netra, and 13 were excluded from the QuickSee device data set due to testing artifacts and patient positioning difficulties.

Of the 190 subjects, 91 were women and 99 were men, and the cohort had an age range of 40 to 88 and a mean age of 52.44 years. With subjective refraction, the range of spherical refractive error was -6.50 Diopters (a unit of measurement of the optical power of a lens or curved mirror, which is equal to the reciprocal of the focal length measured in meters, denoted as “D”) to +6.00 D, with the mean +0.18 D and median +0.50 D. The range of cylinder error was -5.00 to +0.98 D, with the mean -0.58 D and median -0.50 D.

**Fig 1:**
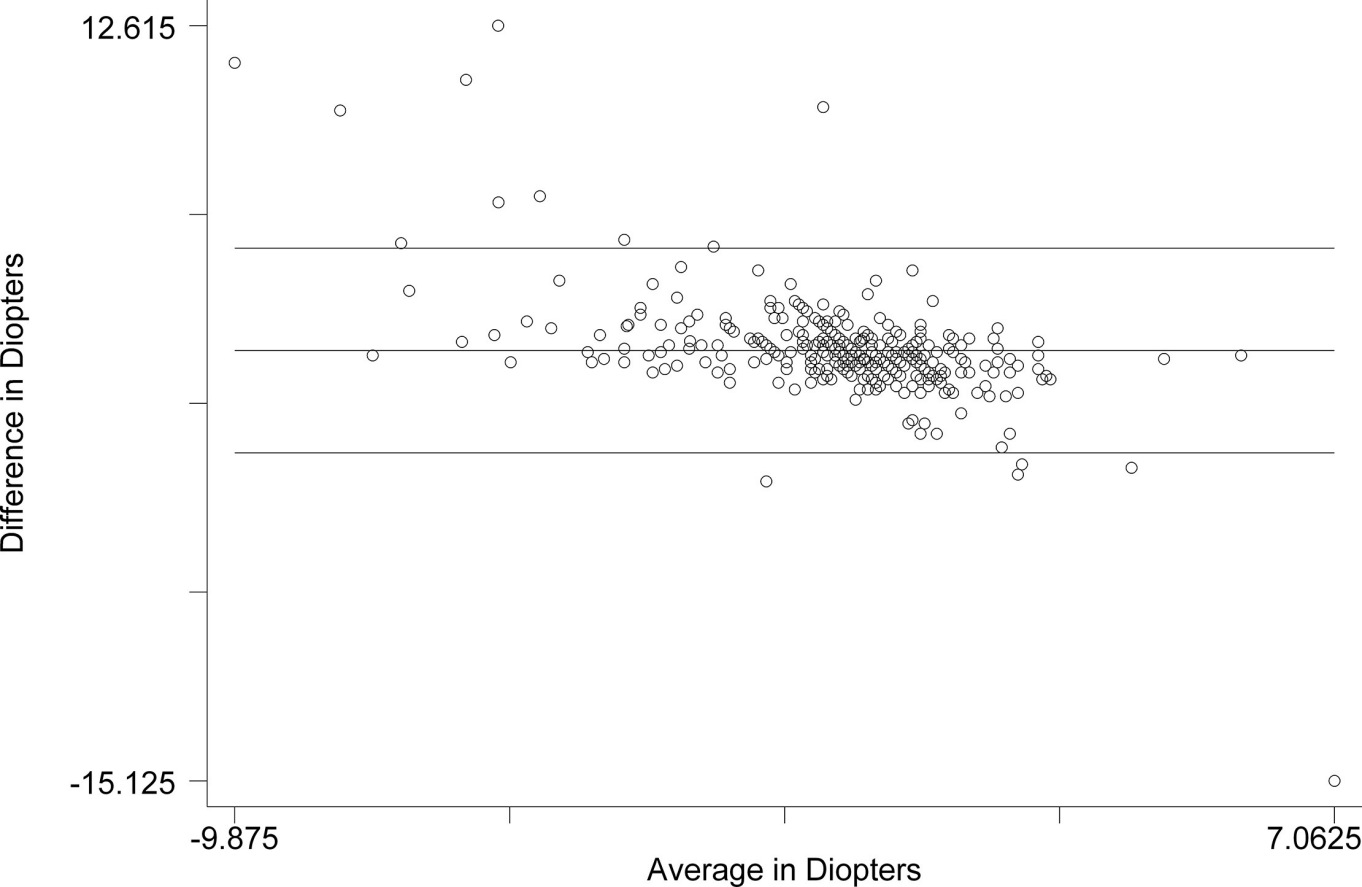
Bland-Altman comparison of spherical equivalent of Subjective Refraction and Retinomax.

The Bland-Altman comparison of Subjective Refraction with Retinomax (Fig 1) revealed the limits of agreement (reference range for difference): -3.076 D to 4.447 D. The mean difference was 0.685 D (CI 0.489 to 0.881). The range was -9.875 D to 7.063 D. The Pitman’s test of difference in variance yielded r = - 0.590, p = 0.000.

**Fig 2:**
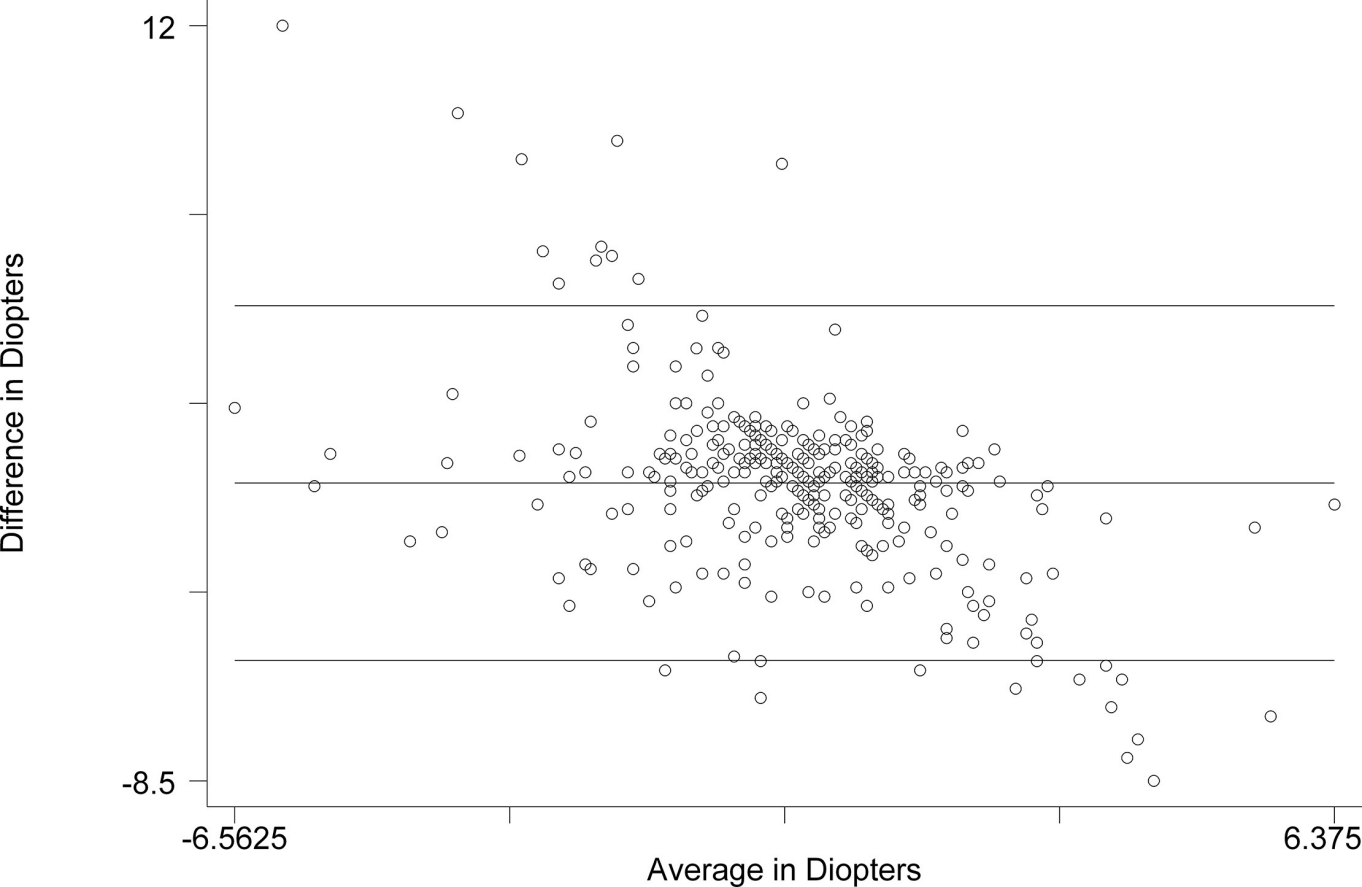
Bland-Altman comparison of spherical equivalent of Subjective Refraction and Netra.

The Bland-Altman comparison of Subjective Refraction with Netra (Fig 2) revealed the limits of agreement: -5.228 D to 4.397 D. The mean difference was -0.416 D (CI -0.675 to -0.157). The range was -6.563 D to 6.375 D. The Pitman’s test of difference in variance yielded r = -0.508, p = 0.000.

**Fig 3:**
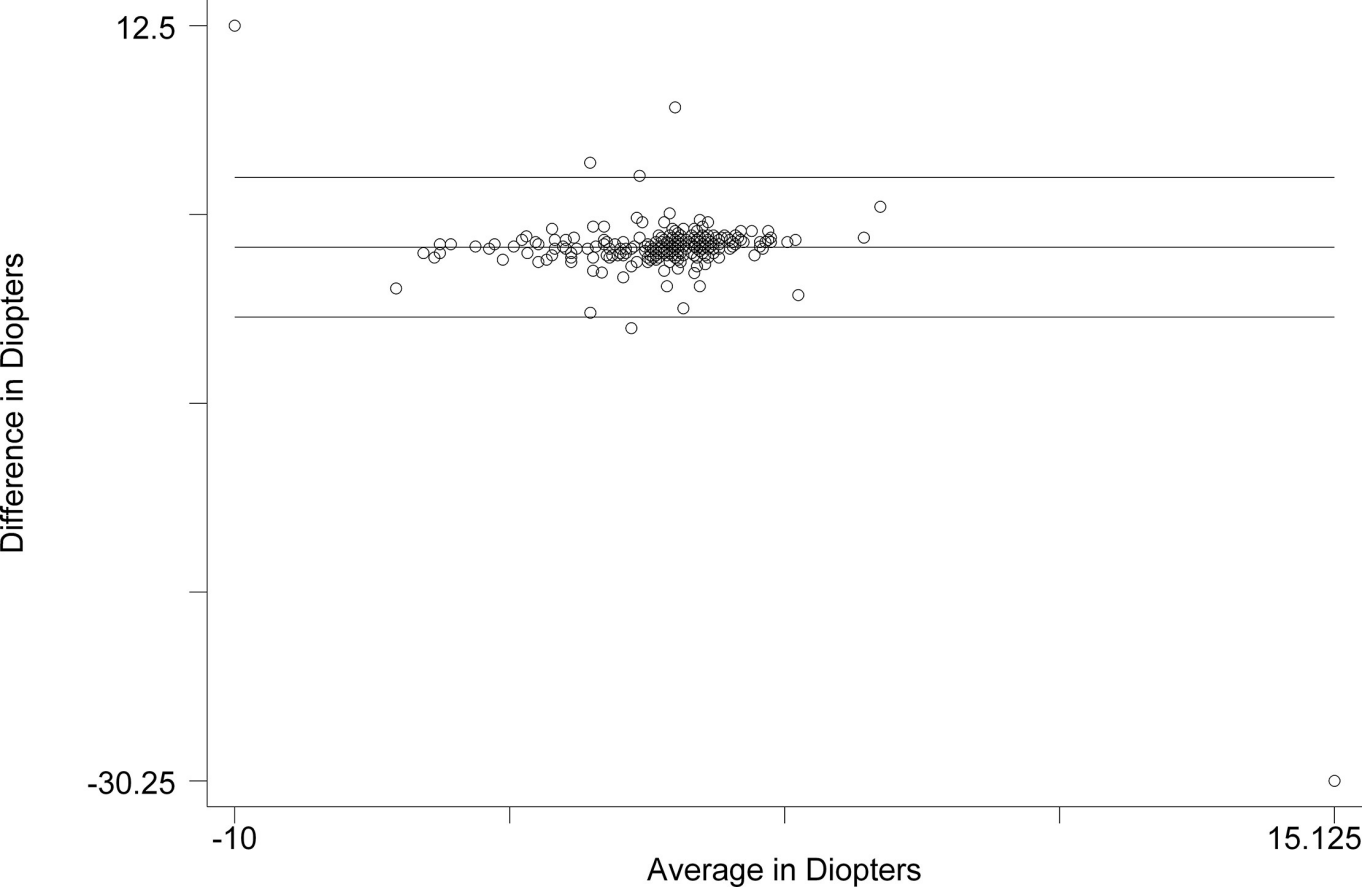
Bland-Altman comparison of spherical equivalent of Subjective Refraction and QuickSee.

The Bland-Altman comparison of Subjective Refraction with QuickSee (Fig 3) revealed the limits of agreement: -3.990 D to 3.912 D. The mean difference was -0.039 D (CI -0.250 to 0.171). The range was -10.000 D to 15.125 D. The Pitman’s test of difference in variance yielded r = -0.440, p = 0.000.

The difference between readings of subjective refraction and handheld autorefractors (both eyes in diopter units) was also analyzed (Table 1).

**Table 1:** Difference between spherical equivalent values for subjective refraction and handheld devices (in Diopters).

Handheld Device	N	Mean	Std. Dev.	Min	Max
Retinomax–Subjective refraction	374	-0.4609626	1.826319	-9.75	17.75
Netra–Subjective refraction	357	0.6257703	2.284967	-13	9.75
QuickSee–Subjective refraction	361	.0163435	0.9309621	-7.5	6.25

The Retinomax had a total N = 374 and a mean of -0.46 D, with a standard deviation of 1.83 D. The Netra had a total N = 357 and a mean of 0.63 D, with a standard deviation of 2.28 D. The QuickSee had a total N = 361 and a mean of 0.02 D, with a standard deviation of 0.93 D.

## Discussion

Of the three mobile autorefractors in this study, the Retinomax device was associated with a short learning curve for both eye-care service providers and study subjects and gave relatively accurate refraction readings. The device took an average of 52 seconds to measure refraction of both the eyes for a respondent. The Retinomax device has also been used in other vision screening studies and found to be a useful tool in diagnosing refractive error.[9] However, it is a delicate instrument and could be damaged in a field study if dropped or otherwise mishandled, requiring costly repair. Furthermore, it retails for US$10,000–$12,000, which makes it particularly expensive for researchers and healthcare providers in low-resource areas, limiting its use in large-scale population surveys and field visits by healthcare providers.

The Netra device is significantly less expensive at US$1,099 and could be a viable and robust lower-budget option. However, in this study it was less accurate and had a long learning curve for both practitioners and subjects. On average, the Netra device took 8 minutes 53 seconds to measure refraction of both the eyes for a respondent. This was in part because the Netra device requires the patient to manipulate dials on the device to align images in the viewing window, an action that patients unaccustomed to using certain types of technology may struggle to grasp. Additionally, use of the Netra requires an Android smartphone, which houses the software programming, to run the associated application, which adds significantly to the total cost of the device.

The QuickSee device is also significantly less expensive than the Retinomax (about 60–75% less—based on the U.S. retail sales price pending at the time of this writing), had a short learning curve for both practitioners and subjects, and had the most accurate measurements of the three handheld devices tested. On average, the QuickSee device took 3 minutes and 11 seconds to measure refraction in both the eyes for a respondent. At the time of this study, the QuickSee had been previously used in field studies in India[10] and had proven efficient and accurate. Its robust construction is an advantage in rural areas and extreme weather conditions, and is also an affordable option for large-scale studies.

The data also reveals that QuickSee and Retinomax, are faster than subjective refraction in measuring refraction, and are somewhat faster than Netra device. It would also be useful to compare the per case costs of these devices, given that they vary in their capital and time costs and possibly their working lives as well.

Assessing and treating uncorrected refractive error in low-resource settings poses several challenges: measuring the refractive error of the population with the use of limited technical resources; obtaining accurate medical diagnoses; and providing solutions such as eye glasses or eye surgery for the impairment. This study demonstrates that refractive error can be accurately measured using relatively inexpensive and portable handheld field devices. Visually impaired people are more likely to be depressed, unemployed, and suffer accidents; women and lower socioeconomic classes experience visual impairment at higher rates than their counterparts, putting them at greater risk for those conditions and compounding the prejudices and challenges these groups already face. The potential benefits of an improvement in the quality of life for individuals currently living with visual impairment due to uncorrected refractive error would also bring significant economic dividends to society, in terms of both reduced healthcare costs and increased productivity for those successfully treated. The deployment of these handheld devices could be an expedient step toward reaching those goals.
